# Fellow-Workers and the Roll of Honour

**Published:** 1915-07-24

**Authors:** 


### ROUND THE WORLD.

[The Editor invites Early Information and Contributions Marked "Fellow-Workers."]

### Fellow-Workers and the Roll of Honour.

THE new hospital for officers at Euston (Endsleigh Palace Hotel) is fortunate in having secured the services of Sir Rickman J. Godlee, who is one of our most eminent consultants, and has for long enjoyed a leading place amongst British surgeons. After many years' work in the wards and operating theatres of University College Hospital, Sir Rickman Godlee was elected to the presidential chair of the Royal College of Surgeons. He was created a baronet in 1912, and last year was made a K.C.V.O.

DR. WILFRED JAMES HADLEY, M.D., F.R.C.P. Lond., also on the staff of the aforementioned hospital for officers, is a well-known member of the staff of the London Hospital, where he occupies the posts of physician, pathologist, and lecturer on medicine. A prolific writer on medical subjects, particularly in connection with lung diseases, Dr. Hadley, who is also physician to the City of London Chest Hospital at Victoria Park, qualified in 1883, and was made an F.R.C.P. in 1899. He is also an F.R.C.S. Eng.

THE vast experience of damaged limbs that has fallen to the lot of surgeons working with the Army has resulted in the collection of much useful data, the study of which has enabled many important modifications of treatment to be made. A series of practical communications in this connection has just been made to the *British Medical Journal* (July 10, 1915) by Colonel H. M. W. Gray, Mr. Norman C. Lake, Captain C. H. Barber, and Mr. E. H. Willock.

COLONEL HENRY M. W. GRAY, who is one of the consulting surgeons attached to the British Army abroad, points out that the experience gained has greatly improved methods of treating wounds of the knee-joint due to rifle-bullets or shell-fire. This in itself is of paramount importance, as permanent damage to the knee-joint is one of the most crippling conditions that can befall a man who wants to earn his living. Colonel Gray is, in civil life, a leading surgeon of Aberdeen, where he holds the posts of surgeon to the Royal Infirmary and to the Royal Hospital for Sick Children. He qualified M.B., C.M. Aberd. in 1895, and subsequently became an F.R.C.S. Edin.

MR. EDWARD HULSE WILLOCK, assistant surgeon to the Croydon General Hospital, has designed an apparatus by which continuous drainage and irrigation of bad limb wounds may be the more effectively carried out. Mr. Willock, who qualified from St. Thomas's in 1891, has worked keenly on behalf of the Croydon division of the British Medical Association.

MR. SIDNEY MAYNARD SMITH, M.B. Lond., F.R.C.S. Eng., is going abroad with a hospital organised by the St. John Ambulance Association. Mr. Maynard Smith, who, it is understood, will have the rank of captain in the R.A.M.C., is surgeon to out-patients at St. Mary's Hospital and surgeon to the London Fever Hospital. Formerly on the staff of the Victoria Hospital for Children, he also saw military service during the South African War, when he went to the Front as a civil surgeon to the Field Force.

OTHER medical men engaged in the public health services who have lately taken up military work include Dr. R. T. Herdman, Dr. C. L. Lakin, Dr. L. L. Preston, and Dr. H. Peck.

DR. RONALD TYDD HERDMAN, M.D., C.M. Edin., D.P.H. Camb., studied medicine at University College Hospital as well as at Edinburgh. Lately he has been school medical officer and deputy medical officer of health for Bedfordshire. Dr. Herdman was for a time assistant house surgeon to the Surrey County Hospital at Guildford, and has also been house surgeon to the Golden Square Throat Hospital.

DR. CECIL LIONEL LAKIN leaves his work as assistant school medical officer for Surrey to take a temporary commission in the R.A.M.C. A Charing Cross Hospital man, he qualified in 1903, and subsequently completing the London University course took the M.D. in State medicine. After holding resident appointments at his hospital, he was for a time assistant medical officer to the South-Eastern Fever Hospital (M.A.B.), and assistant school medical officer for Berkshire.

DR. LIONEL LITCHFIELD PRESTON has obtained leave of absence from his duties as medical officer of health for St. Helens, Isle of Wight. A resident of Ryde, he is surgeon to the Royal Isle of Wight County Hospital and to other local charities. Qualifying from the Middlesex Hospital, he became an M.B., B.S. Durh., and was then for a time clinical assistant to the Central London Ear and Throat Hospital.

THE medical officer of health for Chesterfield, who is now mobilised in the R.A.M.C. (T.F.) as Captain Herbert Peck, has been serving with the 42nd Reserve Park, A.S.C. A barrister-at-law as well as an M.D., C.M. Edin., D.P.H. Cantab., he has given much attention to problems of public health and sanitation, many interesting communications from his pen having appeared in the various technical journals.

MR. ROWLAND WILLIAM COLLUM, the senior anæsthetist to St. Mary's Hospital, will serve with the same unit. One of our leading specialists in the administration of anæsthetics, Mr. Collum was formerly attached to Charing Cross Hospital and to the Hospital for Sick Children, in Great Ormond Street. He served in South Africa as civil medical officer to the Field Force.

AMONGST doctors who have done well as executive Territorial officers is Lieutenant-Colonel Thomas Edward Sandall, who in peace-time practises at Alford, Lincolnshire, and is now on active service in command of the 5th Battalion of the Lincolnshire Regiment. As a medical man he is medical officer of health for Alford and public vaccinator for a local district. Lieutenant-Colonel Sandall, who is a lecturer and examiner for the British Red Cross Society, qualified in 1893 from Charing Cross Hospital by taking the Conjoint Board's diploma, and subsequently became an M.B., B.C. Cantab. His past appointments include those of house physician, house surgeon, and assistant in the electrical department at Charing Cross Hospital.

